# Psychosocial factors, dentist-patient relationships, and oral health-related quality of life: a structural equation modelling

**DOI:** 10.1186/s12955-023-02214-x

**Published:** 2023-12-04

**Authors:** Youngha Song, Liana Luzzi, David Brennan

**Affiliations:** 1 Department of Preventive and Social Dentistry, School of Dentistry, Seoul National University, 101 Daehak-Ro, Jongno-Gu, Seoul, 03080 South Korea; 2 Dental Research Institute, School of Dentistry, Seoul National University, Seoul, South Korea; 3 Australian Research Centre for Population Oral Health, The University of Adelaide, Adelaide, SA Australia

**Keywords:** Oral health, Psychosocial, Dentist-patient relations, Health-related quality of life, South Australia

## Abstract

**Background:**

Psychosocial factors and dentist-patient relationships (DPR) have been suggested to be associated with oral health outcomes. This study aimed to test a conceptual model which hypothesised relationships among psychosocial factors, DPR variables, and oral health-related quality of life (OHRQoL) in the ‘distal-to-proximal’ framework.

**Methods:**

A total of 12,245 adults aged 18 years or over living in South Australia were randomly sampled for the study. Data were collected from self-complete questionnaires in 2015–2016. The outcome variable of Oral Health Impact Profile was used to measure OHRQoL. Psychosocial domain consisted of psychological well-being, social support, and health self-efficacy. DPR domain included trust in dentists, satisfaction with dental care, and dental fear. The hypothesised model was tested using the two-step approach in structural equation modelling.

**Results:**

Data were analysed from 3767 respondents after the screening/preparing process (adjusted valid response rate 37.4%). In the first step of the analysis, confirmatory factor analyses produced acceptable measurement models for each of the six latent variables (GFI = 0.95, CFI = 0.98, RMSEA = 0.04). The final structural model indicated that better well-being, higher self-efficacy, and more satisfaction were associated with lower oral health impact (β = − 0.12, − 0.07, − 0.14, respectively) whereas fear was positively associated (β = 0.19). Among intermediates, support was positively associated with satisfaction within a small effect size (β = 0.06) as compared to self-efficacy with trust (β = 0.22). The invariance of the final model was also confirmed on participants’ SES and dental service characteristics except the variable of ‘last dental visit’.

**Conclusions:**

Psychosocial factors and DPR variables were associated with oral health impact in both direct and indirect paths. The framework of ‘distal-to-proximal’ actions is empirically supported from psychosocial factors via DPR variables to OHRQoL.

**Supplementary Information:**

The online version contains supplementary material available at 10.1186/s12955-023-02214-x.

## Background

At the centre of social epidemiology are social determinants of health such as psychosocial, economic, political, and environmental factors [1, 2]. Among others, psychosocial characteristics have been explored for their close relationships to general and oral health outcomes along with socioeconomic status (SES) [1–3]. A disparate array of variables consisting of the psychosocial factor have been studied in the previous literature. For example, research has adopted psychological well-being, social support, health self-efficacy, personal control and perceived stress for the association with oral health outcomes [2–7].

Dentist-patient relationships (DPR) at clinical encounters are one of the key components of the biopsychosocial model in dentistry [8–10]. The importance of DPR is also acknowledged in the assessment of quality of care and patient-centred care [11], let alone oral health outcomes [9]. Considering the context of clinical encounters, DPR should be integrated into the whole process of dental care [8], coordinating the delivery of actual dental service. Despite the difficulty operationalising the concept of DPR [12], a few relevant constructs have been proposed to assess its multidimensionality such as trust in dentists, satisfaction with dental care, dental fear, therapeutic communication, and involvement in clinical decision making [8, 12–15].

Despite the importance of psychosocial factors and DPR variables, analyses of potential linkages between the two concepts have not been attempted. To explore their interrelationships with oral health outcome, the ‘distal-to-proximal’ framework can provide helpful models. The framework is conceived by the premise that the *distal* and *general* domain (psychosocial factors) is hypothesised to result in oral health outcomes through the *proximal* and dentistry-*specific* domain (DPR variables). For example, social support as a determinant of health can be hypothesised to result in oral health-related quality of life (OHRQoL) via trust in dentists, one of the more proximal variables for the outcome. The initial model tested in this study was established on the framework, with the components of each domain based on the findings of the literature review.

The aim of the study was to examine the conceptual model comprising hypothesised relationships among psychosocial factors, DPR variables, and the oral health outcome. The research question is reflected in the conceptual model: How are psychosocial and DPR factors as explanatory variables related to OHRQoL as an outcome variable? The broad framework of associations drawn in the conceptual diagram was investigated to assess the hypotheses.

## Methods

Ethics approval for this study was granted by the Human Research Ethics Committee of the University of Adelaide (H-288-2011). All procedures in the study were performed in accordance with the Helsinki declaration for ethical standards. Informed consent was implied if participants completed and returned the questionnaires mailed to them.

This cross-sectional data were from the baseline of a wider prospective cohort study, which aimed to analyse the influence of different dental care pathways on changes of oral health outcomes [16]. A total of 12,245 adults aged 18 years or over living in South Australia were randomly sampled from the Electoral Roll in Australia, which is a comprehensive sampling frame since voting is compulsory for eligible Australian adult citizens. Data were collated from self-complete questionnaires by invitees with a primary approach letter and up to four reminders to encourage response in 2015–2016. The sample size was initially calculated from the expected effect size for the original study and considered to be large enough for structural equation models in this study [17].

### Measures

All variables in the analyses were from multi-item psychometric scales, except for a single item of global rating for dental fear. Responses on each item were coded on a five-point Likert scale from 1 (=strongly disagree) to 5 (=strongly agree), except for the Oral Health Impact Profile (OHIP-14) with 0 (=never) to 4 (=very often). Items with a negative statement were included in some scales to prevent acquiescence bias and were reverse-coded for response consistency, such as from 1 to 5 in the corresponding order. Higher scores on a scale indicated better psychosocial and DPR values, aside from higher dental fear and oral health impact. The outcome variable was the OHIP-14 to measure OHRQoL. The OHIP-14 is a 14-item battery of patient-reported oral health outcomes, capturing perceived oral health impact [18]. OHIP-14 has demonstrated acceptable psychometric properties, and is widely used as an oral health-specific measure of quality of life (Cronbach’s α = 0.94 in this study) [19].

The psychosocial domain for the study included psychological well-being, social support, and health self-efficacy. Psychological well-being was quantified using the Satisfaction with Life Scale, which comprises five items reflecting subjective global life satisfaction as a single factor (α = 0.89) [20]. Social support was measured using the Multidimensional Scale of Perceived Social Support with 12 items loaded on three factors: family, friends, and significant others (α = 0.94) [21]. Health self-efficacy was assessed using the Perceived Health Competence Scale, combining outcome and behavioural expectancies from eight items including four reverse-coded items (α = 0.84) [22].

We selected trust in dentists, satisfaction with dental care, and dental fear as potential representatives for the DPR domain [9]. Trust in dentists was assessed using the Dentist Trust Scale validated as a single factor structure with 11 items including three reverse-coded (α = 0.92) [23]. The dental care satisfaction scale was used to measure satisfaction with care received at the last dental visit, a short form of nine items including four reversely coded out of 31-item full scale (α = 0.83) [24]. Dental fear was rated by asking a single question: “Do you feel afraid or distressed when going to the dentist?” (1 = not at all to 5 = extremely afraid), which has been consistently used in national-level surveys in Australia [25].

### Data analysis

Before performing statistical analyses, the collected data were prepared by sorting out the low quality responses and unengaged cases. Respondents with more than 20% of items missing in either scale and/or identical responses to all items in either scale including reverse-coded items were excluded on the criteria. The imputation of missing values for the items of 20% or less in psychometric scales was conducted using the expectation-maximisation algorithm with an iterative maximum likelihood estimation. All samples obtained through the process were randomly split in half to analyse the model with one and cross-validate with the other.

**Fig. 1:**
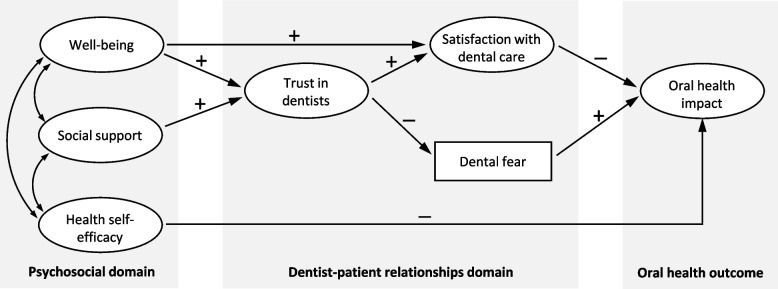
Initial hypothesised conceptual model Shaded boxes indicate each domain in the ‘distal-to-proximal’ framework (error terms not presented); +/− signs for positive/negative associations among variables

The initial conceptual model is drawn in Fig. 1. Each domain rests on the diagram to represent the outline of the ‘distal-to-proximal’ framework. The construction of the initial model was derived from the author’s previous papers about DPR including a pragmatic approach to literature review [13] and relevant findings [9, 14]. Hypotheses of paths to be tested are delineated in the model as straight arrow lines with +/− signs to indicate positive/negative associations among variables. More detailed references for the hypotheses are available in Supplementary Table S1. As we are interested in exploring a vast range of effects and pathways rather than specific estimates of exposures for the population, structural equation modelling (SEM) is advised for this purpose [26]. In particular, we employed the two-step approach in SEM to develop/modify the conceptual model [27]. First, confirmatory factor analyses (CFA) were performed on subsample A to test the validity of measurement models in each domain and an all-inclusive full model. Following the CFA results, the hypothesised model in Fig. 1 was tested for the final structural model. For more general and rigorous results, the final model from subsample A was subjected to further invariance tests of cross-validation with subsample B and multi-group analyses across different groups with participant characteristics (SES and dental service variables) relevant to OHRQoL.

An adequate level of fit indices for measurement and structural models were suggested to be goodness of fit index> 0.95, comparative fit index (CFI) > 0.95, and root mean square error of approximation (RMSEA) < 0.07 [28]. Models were considered to be invariant if the difference of CFI and RMSEA were < 0.01 and < 0.015, respectively [29]. SPSS and AMOS (Versions 25.0., IBM Corp., Chicago, IL, USA) were used for all statistical analyses. Statistical significance was set at *P* < 0.05.

## Results

### Participant characteristics

Data for the analyses were obtained from the final sample of 3767 after excluding 727 participants based on the screening criteria (adjusted valid response rate 37.4%) out of the total 4494 respondents. Sociodemographic and oral health characteristics of the study participants are presented in Table 1. Compared with the population data shown in Supplementary Table S2, the study sample had a higher composition of women (56.0% vs. 50.7%), an older age group (≥60-year-olds of 37.4% vs. 31.8%), and individuals with a post-secondary education (diploma/degree of 40.0% vs. 30.0%). There was no statistical difference in the characteristics between the two half subsamples (Table 1). Mean scores of psychometric scales ranged from 0.5 (SD 0.6) for OHIP to 4.1 (SD 0.9) for social support. Most of each item and sum scores in the scale were within the limit of univariate normality (kurtosis < 7, skewness < 2) [30] except for OHIP which was highly right-skewed. Since multivariate normality could not be assumed from Mardia’s Kurtosis coefficients, bootstrapping with the maximum likelihood method of 2000 times sampling was applied in all SEM analyses [31].

**Table 1:** Sociodemographic and oral health-related characteristics of study participants

Characteristics	Subsample A	Subsample B
	N (valid %)	N (valid %)
**Demographics**		
Sex		
Female	1054 (56.0)	1079 (57.2)
Male	828 (44.0)	806 (42.8)
Age		
18–39	403 (21.4)	428 (22.7)
40–59	775 (41.2)	712 (37.8)
≥ 60	704 (37.4)	745 (39.5)
**Socioeconomic status**		
Income^a^		
< $80,000	990 (57.1)	1012 (58.0)
≥ $80,000	744 (42.9)	734 (42.0)
Education		
≤ Year 12 or certificate	1118 (60.0)	1101 (59.1)
Diploma/degree	746 (40.0)	762 (40.9)
**Oral health behaviours**		
Smoking		
Non-smoker	1655 (88.3)	1667 (88.8)
Smoker	219 (11.7)	211 (11.2)
Tooth brushing		
More than once per day	991 (53.9)	1015 (54.9)
Once per day or less	849 (46.1)	835 (45.1)
**Dental services**		
Last dental visit		
< 12 months	1161 (61.8)	1207 (64.1)
≥ 12 months	718 (38.2)	677 (35.9)
Dental service sector^b^		
Private	1624 (87.2)	1618 (87.6)
Public	238 (12.8)	229 (12.4)
Perceived dental needs		
No	1526 (82.7)	1541 (83.4)
Yes	319 (17.3)	306 (16.6)

^a^Annual income in Australian dollars; ^b^based on the site of the last dental visit

### Confirmatory factor analysis

**Fig. 2:**
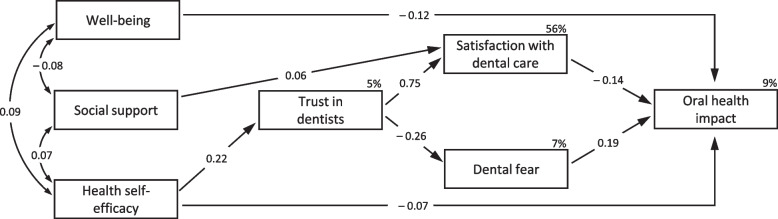
Final structural equation model Path analysis with imputed composite scores for CMB-adjusted values (error terms not presented); *p*-value < 0.01 for all standardised regression weights and correlations on arrow lines; squared multiple correlations on top right edge of each endogenous variable

Model fit indices from CFA on subsample A (*N* = 1882) in each domain and the full measurement model are tabulated in Table 2. All initial models conceived by the original psychometric scales showed unacceptably poor fits from the CFA. Thus, we modified them one-at-a-time according to the following principles: theoretical consideration for less relevant items of the latent variable, mathematical guidance of low factor loadings and modification indices, and invariant item functioning between subsamples. The final full measurement model is drawn in Fig. S1. The model satisfied acceptable fit indices for each domain (upper section in Table 2) and validity/reliability criteria for the CFA (Table S3). All standardised factor loadings in the model were greater than 0.50 with statistical significance (*p* < 0.01). The final measurement model was tested for common method bias (CMB) using the unmeasured latent factor technique [32], which showed differences of standardised regression weights> 0.20 (all well-being items). Hence, we adopted the single-common-method-factor approach [33] for CMB-adjusted values by producing imputed composite scores and applying them to path analysis for the structural model (Fig. 2).

**Table 2:** Model fit indices of structural equation modelling and measurement/structural invariance for cross-validation and multi-group analysis for last dental visit

Model/Invariance	χ^2^	d.f.	χ^2^/d.f.	GFI	CFI	RMSEA [90% CI]
Measurement model^a^						
Psychosocial variables	439.73	71	6.19	0.967	0.981	0.053 [0.048, 0.057]
DPR variables	571.27	75	7.62	0.959	0.981	0.059 [0.055, 0.064]
OHIP-14	53.95	8	6.74	0.991	0.994	0.055 [0.042, 0.070]
Full measurement model	1649.54	507	3.25	0.951	0.979	0.035 [0.033, 0.036]
Structural model^b^						
Initial hypothesised model	167.94	10	16.79	0.975	0.922	0.092 [0.080, 0.104]
Final model	34.31	10	3.43	0.995	0.988	0.036 [0.023, 0.049]
Cross-validation^c^						
Configural invariance	3411.99	1014	3.37	0.949	0.978	0.025 [0.024, 0.026]
Measurement invariance^d^	3452.19	1042	3.31	0.949	0.977	0.025 [0.024, 0.026]
Comparison test^f^	40.20	28			0.001	< 0.001
Configural invariance	151.01	20	7.55	0.988	0.966	0.042 [0.036, 0.048]
Structural invariance^e^	183.40	28	6.55	0.986	0.960	0.038 [0.033, 0.044]
Comparison test^f^	32.39	8			0.006	0.004
Multi-group for last dental visit^g^						
Configural invariance	3363.53	1014	3.32	0.949	0.978	0.025 [0.024, 0.026]
Measurement invariance^d^	3447.04	1042	3.31	0.948	0.977	0.025 [0.024, 0.026]
Comparison test^f^	83.50	28			0.001	< 0.001
Configural invariance	176.36	20	8.82	0.986	0.958	0.046 [0.040, 0.052]
Structural invariance^e^	234.21	28	8.37	0.982	0.944	0.044 [0.039, 0.050]
Comparison test^f^	57.85	8			0.014	0.002

*d.f.* degree of freedom, *GFI* goodness of fit index, *CFI* comparative fit index, *RMSEA* root mean square error of approximation

^a^ Final models from confirmatory factor analysis with subsample A

^b^ Path analysis model with subsample A

^c^ Cross-validation of the final model with subsample B

^d^ Factor loadings constrained equal

^e^ Factor loadings and path coefficients constrained equal

^f^ Difference of χ^2^, d.f., CFI, and RMSEA

^g^ Comparison by multi-group analysis for the time since the last dental visit (within or over 12 months) from all samples

### Structural equation modelling

The initial hypothesised structural model, as shown in Fig. 1, indicated a poor fit to the data (Table 2). Modification of the model was performed with the addition/deletion of paths based on theoretical substantiality and statistical significance one by one until the final model was reached with acceptable fit indices (CFI = 0.99, RMSEA = 0.036). Figure 2 presents the final structural model in the path analysis with all statistically significant coefficients (*p* < 0.01). In the psychosocial domain, well-being and health self-efficacy were negatively associated with oral health impact (β = − 0.12 and − 0.07, respectively). Satisfaction with dental care was negatively (β = − 0.14) associated, while dental fear was positively (β = 0.19) associated with the outcome as direct effects from the DPR domain. Among the intermediates between the two domains, support was positively associated with satisfaction, having a small effect size (β = 0.06) compared to self-efficacy with trust (β = 0.22). Within the DPR domain, trust was associated with satisfaction and fear in different positive/negative directions but with the largest effect sizes (β = 0.75 and − 0.26, respectively). For endogenous variables, the final model explained 9% of the variance in oral health impact; 56, 7, and 5% in satisfaction, fear, and trust, respectively.

### Invariance test

The invariance test results of the final model with cross-validation and multi-group analyses are presented in the lower section of Table 2. The final model was cross-validated on subsample B (*N* = 1885) with the confirmation of configural, measurement, and structural invariances. Different groups with all the participants’ SES and dental service characteristics (Table 1) also produced adequate fit indices for model invariances (Table S4), with the exception of the ‘last dental visit’ variable for the structural invariance (∆CFI = 0.014 in Table 2).

## Discussion

This study tested the hypothesised conceptual model and devised a final structural equation model for the effects of psychosocial factors and DPR variables on oral health impact. The two-step approach in SEM guided modifications of the initial model to the final model with path coefficients for direct and indirect effects, including the mediation of variables on the outcome.

In the first step of SEM, CFA led to measurement models with satisfactory fit indices consisting of each latent variable from each psychometric scale. The results were similar to the findings of previous structural validation between trust and satisfaction with minor variations from different approaches [14]. Reverse-coded items were deleted for low factor loadings from multi-item scales for the acceptable model fit in the first place. Further modifications were predicated on the exclusion of thematically less relevant items and the addition of covariance between analogous items. Those principles were consistently found in CFA for the psychosocial domain, not least self-efficacy, as all items reversely worded were dropped and highly correlated items either deleted or drawn with covariance.

The main concept of the framework, ‘distal-to-proximal’ associations, was supported by the final structural model. Psychosocial factors had indirect effects on oral health impact via DPR variables as mediators, along with their unique contributions of direct effects. The rationale of the ‘proximity’ concept can also be countenanced by the larger effect sizes of DPR variables – the more proximal domain to the outcome. The total effects of DPR variables (|β| from 0.14 to 0.19 in Table S5) were much larger than that of more distal psychosocial factors (|β| from 0.01 to 0.12). This mechanism was also demonstrated within the same DPR domain. Trust, as for the *general* dental context (e.g. trust in general dentists), was entirely mediated by satisfaction and dental fear, as in *specific* clinical settings (e.g. satisfaction with the dental care at the last visit and fear with a descriptive/evocative question of clinical practice) [9]. Therefore, the theory-based framework suggested in the introduction is empirically verified.

For detailed tests of hypotheses and paths of variables, all differences from the initial conceptual model were observed in the psychosocial domain. Well-being was directly associated with OHIP, losing the hypothesised paths to satisfaction and trust. The association of social support was drawn with satisfaction instead of trust initially presumed. Self-efficacy had an additional association with trust in company with a direct effect on OHIP. The positive/negative directions of the paths were all as expected in the hypotheses except for the inverse correlation between well-being and social support. In general, better psychosocial and DPR variables led to lower dental fear and oral health impact. The individual total effects of predictors on the outcome were also in agreement with the findings from the literature review [3–5, 9, 15, 34]. Well-being and self-efficacy were significantly and substantially associated with OHIP (β = − 0.12 and − 0.10 in Table S5), whereas social support was associated with a significant yet negligible amount (β = − 0.01) similar to weak or non-significant results from previous studies [3–5]. Satisfaction and dental fear directly accounted for a considerable amount of variance in OHIP (β = − 0.14 and 0.19, respectively), whereas trust contributed only as an indirect effect. The mediation of trust by satisfaction has already been hypothesised [9] and reported to affect the compliance [35] and loyalty [36] to their physician. Despite its sole indirect associations, trust had a comparable size of the total effect on OHIP (β = − 0.15), which warrants the importance of trust for OHRQoL along with satisfaction and fear.

Multi-group analyses of the final model achieved consistent model invariances across different groups of participant characteristics aside from the ‘last dental visit’ variable. The characteristics in the tests were selected considering the substantial role of SES as a determinant of health [1, 3, 5] and dental service variables for oral health-specific outcomes [3, 5, 9, 15]. For those whose last dental visit was 12 months ago or more, paths with statistical significance in difference showed higher coefficients together with similarly greater β in four paths out of the remaining six (Table S6). Inasmuch as two thirds (65.0% in subsample A) of those whose last visit was less than 12 months ago were for regular check-ups, non-regular dental patients are likely to put more weight on psychosocial and DPR variables for OHRQoL. This claim may need to be verified as a priori hypothesis from this secondary interpretation.

This study has some limitations. First, the direct/indirect effects in the final model need to be interpreted with caution due to the nature of the cross-sectional data. For example, the effect of well-being on OHRQoL can be interpreted in reverse, as those with oral health impact/conditions tend to feel lower satisfaction with life, as reported [37]. Second, a few important variables as either predictors or confounders were missing in the conceptual diagram. Both positive traits and negative aspects of psychosocial factors are supposed to be related with oral health outcomes such as psychological stress [3, 4, 7]. In the DPR domain, communication and patients’ involvement in clinical encounters are considered essential [11, 12] other than those included. Although invariance tests were performed on SES characteristics, income and education may need to be incorporated as functional components in the model for their potential confounding. Next, modified psychometric scales for each latent variable in the CFA may represent slightly different or more specific constructs compared with pre-validated original scales. For example, a modified oral health impact may not comprehensively represent the outcome of the original OHIP-14 by losing some dimensions initially conceived. In this regard, parcelling or total summed scores of items in path analysis can be supplementarily considered for robust results. Finally, data collected entirely from self-complete questionnaires are inherently subject to method biases in empirical studies. Despite our efforts with imputed composite scores to minimise the consequences of CMB, acquiescence bias and social desirability bias might have influenced the results. Also, slight differences in sociodemographic characteristics in the study sample compared to the population data might have resulted in higher trust and satisfaction for females and the elderly, and lower trust for the individuals with higher education, to a limited extent considering the absolute sample size [9]. The results of the study may also need to be interpreted considering these differences.

The findings of the study have several practical implications. The final model shows that psychosocial and DPR values at clinical encounters can contribute to the improvement of oral health outcomes. For example, instead of didactic chairside oral hygiene instructions, a programme to establish a trustful relationship in dental encounters and improve oral health literacy for patients’ health self-efficacy can be more beneficial. Subjective psychosocial factors may need to be considered as much as objective socioeconomic determinants to understand the social gradient of health [3]. This can be vindicated by the common risk factor approach [38] that psychosocial values can be applicable to extensive social milieu as determinants beyond oral and general health. Further studies are advised to establish rigorous causality in a longitudinal design and the general application of the findings to different/diverse outcomes in relevant fields.

## Conclusions

This study found that psychosocial factors and DPR variables are associated with oral health impact in both direct and indirect paths. The framework of ‘distal-to-proximal’ actions is empirically supported from psychosocial factors via DPR variables to OHRQoL. The theoretical biopsychosocial model of health is practically encouraged for improved health promotion, not least for self-reported health outcomes, with the importance of subjective psychosocial determinants.

### Supplementary Information

**Additional file 1.** Figure S1 and Tables S1-S6 for supplementary results of analyses

## Data Availability

The datasets generated and/or analysed during the current study are not publicly available due to privacy or ethical restrictions but are available from the corresponding author on reasonable request.

## Abbreviations

DPR: dentist-patient relationships
OHRQoL: oral health-related quality of life
SES: socioeconomic status
OHIP-14: Oral Health Impact Profile
SEM: structural equation modelling
CFA: confirmatory factor analyses
CMB: common method bias

## References

[1] Watt RG (2007). From victim blaming to upstream action: tackling the social determinants of oral health inequalities. Community Dent Oral Epidemiol.

[2] Sanders AE (2007). Social determinants of oral health: conditions linked to socioeconomic inequalities in oral health in the Australian population: Australian Institute of Health and Welfare.

[3] Brennan DS, Spencer AJ, Roberts-Thomson KF (2019). Socioeconomic and psychosocial associations with oral health impact and general health. Community Dent Oral Epidemiol.

[4] Brennan DS, Mittinty MM, Jamieson L (2019). Psychosocial factors and self-reported transitions in oral and general health. Eur J Oral Sci.

[5] Armfield JM, Mejía GC, Jamieson LM (2013). Socioeconomic and psychosocial correlates of oral health. Int Dent J.

[6] Brennan DS, Spencer A (2012). Social support and optimism in relation to the oral health of young adults. Int J Behav Med.

[7] Sanders AE, Slade GD, Turrell G, Spencer AJ, Marcenes W (2007). Does psychological stress mediate social deprivation in tooth loss?. J Dent Res.

[8] Yamalik N (2005). Dentist-patient relationship and quality care 1. Introduction Int Dent J.

[9] Song Y, Luzzi L, Chrisopoulos S, Brennan D (2020). Dentist-patient relationships and oral health impact in Australian adults. Community Dent Oral Epidemiol.

[10] Bedos C, Apelian N, Vergnes J-N (2018). Social dentistry: an old heritage for a new professional approach. Br Dent J.

[11] Committee on Quality of Health Care in America (2001). Crossing the quality chasm: a new health system for the 21st century.

[12] Hoff T, Collinson GE (2017). How do we talk about the physician–patient relationship? What the nonempirical literature tells us. Med Care Res Rev.

[13] Song Y, Luzzi L, Brennan DS (2020). Trust in dentist-patient relationships: mapping the relevant concepts. Eur J Oral Sci.

[14] Song Y, Luzzi L, Chrisopoulos S, Brennan D (2020). Are trust and satisfaction similar in dental care settings?. Community Dent Oral Epidemiol.

[15] Muirhead VE, Marcenes W, Wright D (2014). Do health provider-patient relationships matter? Exploring dentist-patient relationships and oral health-related quality of life in older people. Age Ageing.

[16] Australian Research Centre for Population Oral Health. Dental Care and Oral Health Study 2018, Available from: https://www.adelaide.edu.au/arcpoh/dentalcarestudy/.

[17] Wolf EJ, Harrington KM, Clark SL, Miller MW (2013). Sample size requirements for structural equation models: an evaluation of power, bias, and solution propriety. Educ Psychol Meas.

[18] Slade GD (1997). Derivation and validation of a short-form oral health impact profile. Community Dent Oral Epidemiol.

[19] Brennan DS (2013). Oral health impact profile, EuroQol, and assessment of quality of life instruments as quality of life and health-utility measures of oral health. Eur J Oral Sci.

[20] Diener E, Emmons RA, Larsen RJ, Griffin S (1985). The satisfaction with life scale. J Pers Assess.

[21] Dahlem NW, Zimet GD, Walker RR (1991). The multidimensional scale of perceived social support: a confirmation study. J Clin Psychol.

[22] Smith MS, Wallston KA, Smith CA (1995). The development and validation of the perceived health competence scale. Health Educ Res.

[23] Armfield J, Ketting M, Chrisopoulos S, Baker S (2017). Do people trust dentists? Development of the dentist trust scale. Aust Dent J.

[24] Stewart J, Spencer A (2005). Dental satisfaction survey 2002.

[25] Armfield JM, Slade GD, Spencer AJ (2009). Dental fear and adult oral health in Australia. Community Dent Oral Epidemiol.

[26] VanderWeele TJ (2012). Structural equation models and epidemiologic analysis. Am J Epidemiol.

[27] Anderson JC, Gerbing DW (1988). Structural equation modeling in practice: a review and recommended two-step approach. Psychol Bull.

[28] Hooper D, Coughlan J, Mullen M (2008). Structural equation modelling: guidelines for determining model fit. Electron J Bus Res Methods.

[29] Chen FF (2007). Sensitivity of goodness of fit indexes to lack of measurement invariance. Struct Equ Model Multidiscip J.

[30] Curran PJ, West SG, Finch JF (1996). The robustness of test statistics to nonnormality and specification error in confirmatory factor analysis. Psychol Methods.

[31] Byrne BM (2010). Structural equation modeling with AMOS : basic concepts, applications, and programming. 2nd ed.

[32] Jordan PJ, Troth AC (2020). Common method bias in applied settings: the dilemma of researching in organizations. Aust J Manag.

[33] Podsakoff PM, MacKenzie SB, Lee J-Y, Podsakoff NP (2003). Common method biases in behavioral research: a critical review of the literature and recommended remedies. J Appl Psychol.

[34] Mehrstedt M, John MT, Tönnies S, Micheelis W (2007). Oral health-related quality of life in patients with dental anxiety. Community Dent Oral Epidemiol.

[35] Kim SS, Kaplowitz S, Johnston MV (2004). The effects of physician empathy on patient satisfaction and compliance. Eval Health Prof.

[36] Platonova EA, Kennedy KN, Shewchuk RM (2008). Understanding patient satisfaction, trust, and loyalty to primary care physicians. Med Care Res Rev.

[37] Brennan DS, Spencer AJ, Roberts-Thomson KF (2008). Tooth loss, chewing ability and quality of life. Qual Life Res.

[38] Sheiham A, Watt RG (2000). The common risk factor approach: a rational basis for promoting oral health. Community Dent Oral Epidemiol.

