# Supplementary material for: Psychosocial factors, dentist-patient relationships, and oral health-related quality of life: a structural equation modelling
Source: Health Qual Life Outcomes. 2023 Dec 4;21:130. doi: 10.1186/s12955-023-02214-x (PMC10696761; doi:10.1186/s12955-023-02214-x)
Supplement: Supplementary file 1 — Additional file 1. Figure S1 and Tables S1-S6 for supplementary results of analyses [file 12955_2023_2214_MOESM1_ESM.docx]

Supplementary material

**Psychosocial factors, dentist-patient relationships, and oral health-related quality of life: a structural equation modelling**

Youngha Song, Liana Luzzi, David Brennan

Table S1. References for the construction of the initial conceptual model ……… 2

Table S2. Study participants’ sociodemographic characteristics ………………… 3

Figure S1. Full measurement model of confirmatory factor analysis ……………… 4

Table S3. Validity and reliability of full model in confirmatory factor analysis …… 5

Table S4. Invariance tests from multi-group analyses ……………………………… 6

Table S5. Total, direct, and indirect effect in the final path analysis model ………… 7

Table S6. Path coefficients from multi-group analysis for ‘last dental visit’ ………… 8

**Table S1**. References for the construction of the initial conceptual model

| **Domain** | **Variables** | **References** |
| --- | --- | --- |
| psychosocial |  | Brennan DS, Mittinty MM, Jamieson L. Psychosocial factors and self‐reported transitions in oral and general health. Eur J Oral Sci. 2019.  Armfield JM, Mejía GC, Jamieson LM. Socioeconomic and psychosocial correlates of oral health. International dental journal. 2013;63(4):202-9. |
|  | Well-being | Brennan DS, Spencer AJ, Roberts-Thomson KF. Tooth loss, chewing ability and quality of life. Quality of Life Research. 2008;17(2):227-35.  Brennan DS, Keuskamp D, Balasubramanian M, Amarasena N. General health and well-being among primary care patients aged 75+ years: Associations with living conditions, oral health and dependency. Australas J Ageing. 2018;37(1):E1-E6. Epub 2017/11/14. |
|  | Social support | Ommen O, Thuem S, Pfaff H, Janssen C. The relationship between social support, shared decision-making and patient's trust in doctors: a cross-sectional survey of 2,197 inpatients using the Cologne Patient Questionnaire. Int J Public Health. 2011;56(3):319-27.  Brennan DS, Mittinty MM, Jamieson L. Psychosocial factors and self‐reported transitions in oral and general health. Eur J Oral Sci. 2019. |
|  | Health self-efficacy | Brennan DS, Spencer AJ, Roberts‐Thomson KF. Socioeconomic and psychosocial associations with oral health impact and general health. Community Dentistry and Oral Epidemiology. 2019;47(1):32-9.  Parker EJ, Haag DG, Spencer AJ, Roberts-Thomson K, Jamieson LM. Self-efficacy and oral health outcomes in a regional Australian Aboriginal population. BMC Oral Health. 2022 Oct 17;22(1):447. |
| Dentist-patient relationships |  | Muirhead VE, Marcenes W, Wright D. Do health provider-patient relationships matter? Exploring dentist-patient relationships and oral health-related quality of life in older people. Age and Ageing. 2014;43(3):399-405.  Song Y, Luzzi L, Brennan DS. Trust in dentist-patient relationships: mapping the relevant concepts. Eur J Oral Sci. 2020;128(2):110-9. |
|  | Trust in dentists | Subramanian SV, Kim DJ, Kawachi I. Social trust and self-rated health in US communities: a multilevel analysis. Journal of Urban Health. 2002;79(1):S21-S34.  Song Y, Luzzi L, Chrisopoulos S, Brennan D. Dentist-patient relationships and oral health impact in Australian adults. Community Dentistry and Oral Epidemiology. 2020;48(4):309-16.  Song Y, Luzzi L, Chrisopoulos S, Brennan D. Are trust and satisfaction similar in dental care settings? Community Dent Oral Epidemiol. 2020;48(6):480-6. |
|  | Satisfaction with dental care | Luo JYN, Liu PP, Wong MCM. Patients’ satisfaction with dental care: a qualitative study to develop a satisfaction instrument. BMC Oral Health. 2018;18(1):15.  Balkrishnan R, Dugan E, Camacho FT, Hall MA. Trust and satisfaction with physicians, insurers, and the medical profession. Medical Care. 2003;41(9):1058-64.  Song Y, Luzzi L, Chrisopoulos S, Brennan D. Dentist-patient relationships and oral health impact in Australian adults. Community Dentistry and Oral Epidemiology. 2020;48(4):309-16.  Song Y, Luzzi L, Chrisopoulos S, Brennan D. Are trust and satisfaction similar in dental care settings? Community Dent Oral Epidemiol. 2020;48(6):480-6. |
|  | Dental fear | Meng X, Heft MW, Bradley MM, Lang PJ. Effect of fear on dental utilization behaviors and oral health outcome. Community dentistry and oral epidemiology. 2007 Aug;35(4):292-301.  Bell RA, Arcury TA, Anderson AM, Chen H, Savoca MR, Gilbert GH, Quandt SA. Dental anxiety and oral health outcomes among rural older adults. Journal of public health dentistry. 2012 Jan;72(1):53-9.  Song Y, Luzzi L, Chrisopoulos S, Brennan D. Dentist-patient relationships and oral health impact in Australian adults. Community Dentistry and Oral Epidemiology. 2020;48(4):309-16. |

**Table S2**. Study participants’ sociodemographic characteristics in percentage and comparison with population data

|  | Data from 2016 Census^a^ (%) | Distribution in the study^b^ (95% CI) | Distribution of excluded cases^c^ (95% CI) |
| --- | --- | --- | --- |
| Sex |  |  |  |
| Female | 50.7^­0^ | 56.0 (53.8-58.2) | 53.0 (49.3-56.6) |
| Male | 49.3^­0^ | 44.0 (41.8-46.2) | 47.0 (43.4-50.7) |
| Age |  |  |  |
| 18–39 | 33.4^¶^ | 21.4 (19.6-23.3) | 15.8 (13.3-18.6) |
| 40–59 | 34.7^­0^ | 41.2 (39.0-43.4) | 32.0 (28.7-35.5) |
| ≥60 | 31.8^­0^ | 37.4 (35.2-39.6) | 52.1 (48.5-55.7) |
| Income (annual household in AUD) |  |  |  |
| <$80,000 | 60.2^§^ | 57.1 (54.8-59.4) | 72.1 (68.3-75.6) |
| ≥$80,000 | 39.8^­0^ | 42.9 (40.6-45.2) | 27.9 (24.4-31.7) |
| Education |  |  |  |
| ≤Year 12 or certificate | 70.0^­0^ | 60.0 (57.7-62.2) | 69.6 (66.0-73.0) |
| Diploma/degree | 30.0^­0^ | 40.0 (37.8-42.3) | 30.4 (27.0-34.0) |
| Dental service sector |  |  |  |
| Private | 88.5^†^ | 87.2 (85.6-88.7) | 80.2 (77.1-83.1) |

^a^ 2016 Census: South Australia (from the Australian Bureau of Statistics <https://quickstats.censusdata.abs.gov.au/census_services/getproduct/census/2016/quickstat/4>); ^b^ Characteristics of study participants in subsample A; ^c^ Characteristics of excluded 727 participants based on the screening criteria; ^¶^ Age 20-39; ^§^ <$78,000 (<$1,500/week); ^†^ dentate people aged 15 and over from AIHW: Chrisopoulos S, Harford JE & Ellershaw A 2016. Oral health and dental care in Australia: key facts and figures 2015. Cat. no. DEN 229. Canberra: AIHW.


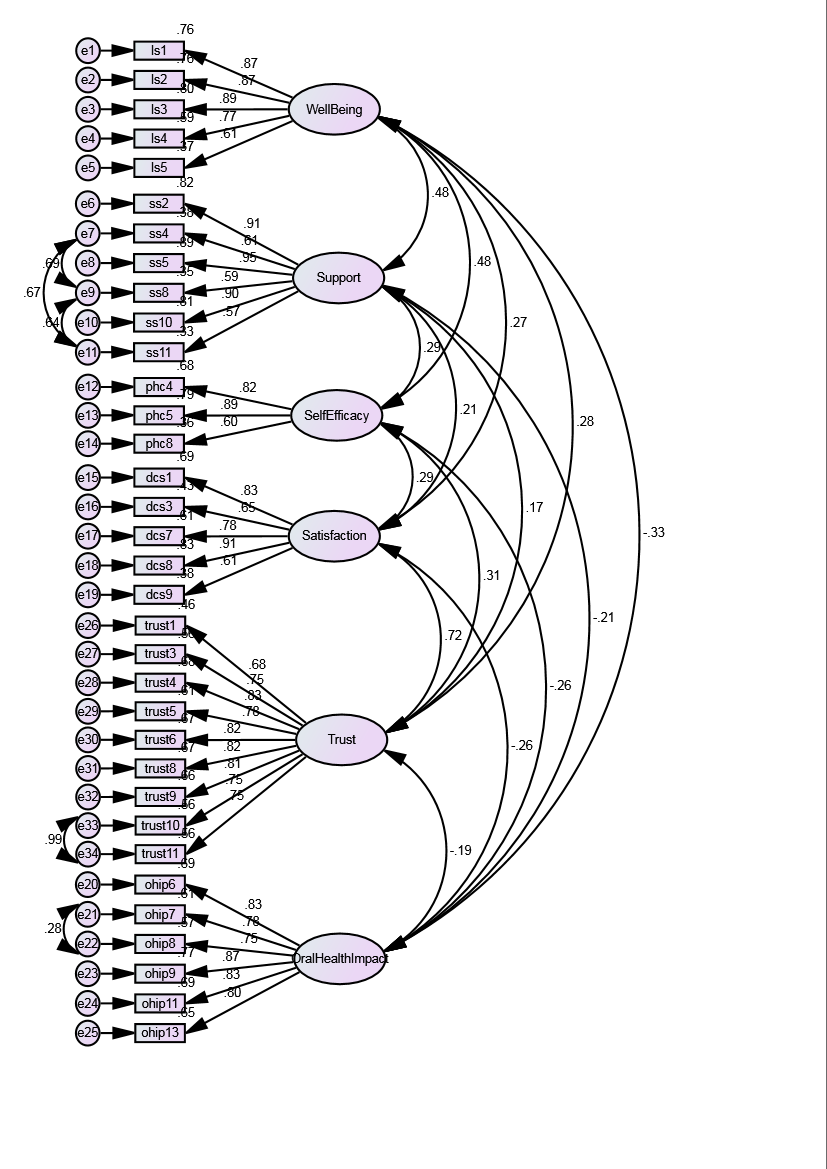
**Figure S1.** Full measurement model of confirmatory factor analysis

p-value <0.01 for all standardised regression weights and correlations on arrow lines; squared multiple correlations on arrow heads; ls, Satisfaction With Life Scale; ss, Perceived Social Stress; phc, Perceived Health Competence Scale; dcs, Dental Care Satisfaction; trust, Dentist Trust Scale; ohip, Oral Health Impact Profile

**Table S3**. Validity and reliability of full model in confirmatory factor analysis

|  | SWL | PSS | PHC | DTS | DCS | OHIP |  | AVE | CR | N | α |
| --- | --- | --- | --- | --- | --- | --- | --- | --- | --- | --- | --- |
| SWL | **0.811** |  |  |  |  |  |  | 0.657 | 0.904 | 5 | 0.895 |
| PSS | 0.482 | **0.773** |  |  |  |  |  | 0.597 | 0.895 | 6 | 0.903 |
| PHC | 0.476 | 0.287 | **0.779** |  |  |  |  | 0.607 | 0.819 | 3 | 0.802 |
| DTS | 0.280 | 0.174 | 0.307 | **0.777** |  |  |  | 0.603 | 0.932 | 9 | 0.934 |
| DCS | 0.272 | 0.206 | 0.291 | 0.724 | **0.766** |  |  | 0.587 | 0.874 | 5 | 0.855 |
| OHIP | -0.331 | -0.206 | -0.256 | -0.194 | -0.257 | **0.813** |  | 0.661 | 0.921 | 6 | 0.920 |

Correlations between factors with root square of AVE as boldface; SWL, Satisfaction With Life Scale; PSS, Perceived Social Stress; PHC, Perceived Health Competence Scale; DTS, Dentist Trust Scale; DCS, Dental Care Satisfaction; OHIP, Oral Health Impact Profile; AVE, Average Variance Extracted; CR, Composite Reliability; N, number of items; α, Cronbach’s α

**Table S4**. Invariance tests from multi-group analyses

| Model | χ^2^ | d.f. | CMIN/d.f. | GFI | CFI | RMSEA [90% CI] |
| --- | --- | --- | --- | --- | --- | --- |
| Multi-group for Income |  |  |  |  |  |  |
| Configural invariance | 3158.58 | 1014 | 3.12 | .949 | .978 | .025 [.024, .026] |
| Measurement invariance | 3351.82 | 1042 | 3.22 | .946 | .976 | .025 [.024, .026] |
| Comparison test | 193.24 | 28 |  |  | .002 | <.001 |
| Configural invariance | 157.51 | 20 | 7.88 | .987 | .962 | .044 [.038, .051] |
| Structural invariance | 184.36 | 28 | 6.58 | .984 | .957 | .040 [.035, .046] |
| Comparison test | 26.85 | 8 |  |  | .005 | .004 |
| Multi-group for Education |  |  |  |  |  |  |
| Configural invariance | 3310.86 | 1014 | 3.27 | .950 | .978 | .025 [.024, .026] |
| Measurement invariance | 3437.42 | 1042 | 3.30 | .948 | .977 | .025 [.024, .026] |
| Comparison test | 126.56 | 28 |  |  | .001 | <.001 |
| Configural invariance | 173.19 | 20 | 8.66 | .986 | .961 | .045 [.039, .052] |
| Structural invariance | 180.14 | 28 | 6.43 | .986 | .961 | .038 [.033, .044] |
| Comparison test | 6.96 | 8 |  |  | <.001 | .007 |
| Multi-group for Dental service sector |  |  |  |  |  |  |
| Configural invariance | 3324.09 | 1014 | 3.28 | .950 | .978 | .025 [.024, .026] |
| Measurement invariance | 3376.27 | 1042 | 3.24 | .949 | .978 | .025 [.024, .026] |
| Comparison test | 52.18 | 28 |  |  | <.001 | <.001 |
| Configural invariance | 179.97 | 20 | 9.00 | .986 | .959 | .046 [.040, .053] |
| Structural invariance | 196.78 | 28 | 7.03 | .985 | .957 | .040 [.035, .046] |
| Comparison test | 16.81 | 8 |  |  | .002 | .006 |
| Multi-group for Dental needs |  |  |  |  |  |  |
| Configural invariance | 3241.31 | 1014 | 3.20 | .951 | .978 | .024 [.023, .025] |
| Measurement invariance | 3303.11 | 1042 | 3.17 | .950 | .978 | .024 [.023, .025] |
| Comparison test | 61.80 | 28 |  |  | <.001 | <.001 |
| Configural invariance | 170.84 | 20 | 8.54 | .987 | .959 | .045 [.039, .052] |
| Structural invariance | 204.99 | 28 | 7.32 | .984 | .952 | .041 [.036, .047] |
| Comparison test | 34.15 | 8 |  |  | .007 | .004 |

d.f., degree of freedom; GFI, goodness of fit index; CFI, comparative fit index; RMSEA, root mean square error of approximation

Comparison by multi-group analysis for the variable of participants’ characteristic from all samples; Measurement invariance, Factor loadings constrained equal; Structural invariance, Factor loadings and path coefficients constrained equal; Comparison test, Difference of χ^2^, d.f., CFI, and RMSEA

**Table S5**. Total, direct, and indirect effect with standardised estimates in the final path analysis model

|  |  | SWL | PSS | PHC | DTS | Fear | DCS |
| --- | --- | --- | --- | --- | --- | --- | --- |
| DTS |  |  |  |  |  |  |  |
|  | Total |  |  | 0.223 (0.025) |  |  |  |
|  | Directlll |  |  | 0.223 (0.025) |  |  |  |
|  | Indirect |  |  |  |  |  |  |
| Fear |  |  |  |  |  |  |  |
|  | Total |  |  | -0.058 (0.008) | -0.258 (0.024) |  |  |
|  | Directlll |  |  |  | -0.258(0.024) |  |  |
|  | Indirect |  |  | -0.058 (0.008) |  |  |  |
| DCS |  |  |  |  |  |  |  |
|  | Total |  | 0.055 (0.016) | 0.167 (0.019) | 0.749 (0.013) |  |  |
|  | Directlll |  | 0.055 (0.016) |  | 0.749 (0.013) |  |  |
|  | Indirect |  |  | 0.167 (0.019) |  |  |  |
| OHIP |  |  |  |  |  |  |  |
|  | Total | -0.119 (0.024) | -0.008 (0.003) | -0.099 (0.028) | -0.150 (0.021) | 0.185 (0.029) | -0.136 (0.027) |
|  | Directlll | -0.119 (0.024) |  | -0.066 (0.027) |  | 0.185 (0.029) | -0.136 (0.027) |
|  | Indirect |  | -0.008 (0.003) | -0.033 (0.006) | -0.150 (0.021) |  |  |

p-value <0.01 for all standardised estimates; Bootstrapped standard errors in parentheses; SWL, Satisfaction With Life Scale; PSS, Perceived Social Stress; PHC, Perceived Health Competence Scale; DTS, Dentist Trust Scale; DCS, Dental Care Satisfaction; OHIP, Oral Health Impact Profile

**Table S6**. Standardised path coefficients from multi-group analysis for the characteristic of ‘last dental visit’

|  |  |  | < 12 months |  |  | ≥ 12 months |  |  |  |
| --- | --- | --- | --- | --- | --- | --- | --- | --- | --- |
|  |  |  | β | P value |  | β | P value |  | z-score |
| PHC | → | DTS | .171 | <0.001 |  | .184 | <0.001 |  | 0.613 |
| DTS | → | DCS | .708 | <0.001 |  | .755 | <0.001 |  | **6.687** |
| DTS | → | fear | -.210 | <0.001 |  | -.211 | <0.001 |  | -0.652 |
| PSS | → | DCS | .042 | 0.004 |  | .038 | 0.029 |  | 0.150 |
| fear | → | OHIP | .190 | <0.001 |  | .164 | <0.001 |  | -0.903 |
| DCS | → | OHIP | -.124 | <0.001 |  | -.168 | <0.001 |  | -1.067 |
| PHC | → | OHIP | -.038 | 0.056 |  | -.070 | 0.006 |  | -1.182 |
| SWL | → | OHIP | -.111 | <0.001 |  | -.201 | <0.001 |  | **-3.038** |

β, standardised regression weights from multi-group analysis for the time since the last dental visit (within or over 12 months) from all samples; z-score, significant differences are marked in bold face (p<0.01); SWL, Satisfaction With Life Scale; PSS, Perceived Social Stress; PHC, Perceived Health Competence Scale; DTS, Dentist Trust Scale; DCS, Dental Care Satisfaction; OHIP, Oral Health Impact Profile
